# New trends in articular cartilage repair

**DOI:** 10.1186/s40634-015-0026-0

**Published:** 2015-04-02

**Authors:** Magali Cucchiarini, Christel Henrionnet, Didier Mainard, Astrid Pinzano, Henning Madry

**Affiliations:** Center of Experimental Orthopaedics, Saarland University Medical Center and Saarland University, Bldg 37, Kirrbergerstr. 1, D-66421 Homburg, Germany; Cartilage Net of the Greater Region, Homburg, Germany; Ingénierie Moléculaire et Physiopathologie Articulaire, Unité Mixte de Recherches 7365 Centre National de la Recherche Scientifique, Université de Lorraine, F-54505 Vandoeuvre Lès Nancy, France; Department of Orthopaedic Surgery, Saarland University Medical Center and Saarland University, D-66421 Homburg/Saar, Germany

**Keywords:** Cartilage repair, Bioengineering, Non-invasive second harmonic generation imaging, Stem cells, rAAV gene therapy, Osteoarthritis, Infrapatellar fat pad

## Abstract

Damage to the articular cartilage is an important, prevalent, and unsolved clinical issue for the orthopaedic surgeon. This review summarizes innovative basic research approaches that may improve the current understanding of cartilage repair processes and lead to novel therapeutic options. In this regard, new aspects of cartilage tissue engineering with a focus on the choice of the best-suited cell source are presented. The importance of non-destructive cartilage imaging is highlighted with the recent availability of adapted experimental tools such as Second Harmonic Generation (SHG) imaging. Novel insights into cartilage pathophysiology based on the involvement of the infrapatellar fat pad in osteoarthritis are also described. Also, recombinant adeno-associated viral vectors are discussed as clinically adapted, efficient tools for potential gene-based medicines in a variety of articular cartilage disorders. Taken as a whole, such advances in basic research in diverse fields of articular cartilage repair may lead to the development of improved therapies in the clinics for an improved, effective treatment of cartilage lesions in a close future.

## Introduction

Articular cartilage damage is an important, prevalent, and unsolved clinical issue following joint trauma or during osteoarthritis (OA). In the absence of vascularity, the cartilage does not have access to progenitor cells that may support and participate in regenerative processes. Clinical interventions such as marrow stimulation techniques that support cell invasion from the bone marrow do not restore the original cartilage structure and function in the lesions. Instead, these options lead to the formation of a poorly organized, mechanically inadapted fibrocartilage made of type-I collagen instead of type-II collagen with proteoglycans that are normally found in the hyaline cartilage (Johnstone et al. 2013). Furthermore, such fibrocartilaginous repair tissue may not integrate well with the surrounding, unaffected cartilage (Khan et al. 2008), and may induce the development of osteoarthritis over time (Schinhan et al. 2012).

New, effective treatments are thus needed to enhance the intrinsic repair capacities of injured articular cartilage that might be envisaged based on a better understanding of the pathological events underlying cartilage degradation. In this regard, original findings from active basic and applied experimental research have emerged that may allow to elaborate novel concepts for therapy. They include the identification of new components and tissues involved in the pathological processes of cartilage damage and the development of cell-, gene-, and tissue engineered-based approaches that may positively influence the protective and reparative activities of this highly specialized tissue in sites of injury.

## Review

### Bioengineering and cartilage

Cartilage tissue engineering is the creation of functional substitutes of native cartilage by attaching cells with a chondrogenic potential to polymer scaffolds. Once generated and tested *in vitro*, such constructs might be directly implanted in sites of cartilage injury in the patient, especially in the case of well circumscribed (focal) lesions. The three-dimensional (3D) environment of a scaffold is crucial in cartilage engineering strategies for cell entrapment, proliferation, and chondrogenic differentiation. A scaffold must display the several, following features: they must be biocompatible, must allow for cell adhesion and proliferation, and preferably be biodegradable. Various biodegradable scaffolds based on natural or synthetic polymers have been developed for cartilage tissue engineering (Madry et al. 2002; Sohier et al. 2007; Henrionnet et al. 2010; Henrionnet et al. 2012; Huot et al. 2013; Tritz-Schiavi et al. 2010; Heiligenstein et al. 2011b; Heiligenstein et al. 2011a; Madry et al. 2013; Madry et al. 2014; Rey-Rico et al. 2014b). The importance of the scaffold microarchitecture has been demonstrated for articular cartilage repair (Matsiko et al. 2014).

Chondrocytes may provide the best source of cells for cartilage engineering. Due to the relatively low cellularity of the cartilage, *in vitro* expansion phases are necessary to obtain a sufficient number of cells. Yet, expansion of chondrocytes leads to rapid cell dedifferentiation and consequently to the loss of the chondrogenic phenotype (Benya et al. 1978). Alternative cell sources include embryonic stem cells (ESCs), inducible pluripotent stem cells (iPSCs), and mesenchymal stem cells (MSCs) (de Isla et al. 2010; Stoltz et al. 2010; Orth et al. 2014). ESCs are pluripotent cells derived from the blastocyst but their use is still limited by the risk of teratoma formation and by ethical concerns. iPSCs are other promising cells for clinical applications but again, a risk of tumorigenicity has been identified with their use (Yamashita et al. 2013). MSCs are an alternative valid source of cells for cartilage regeneration as they can be easily gained and expanded *in vitro* without losing their differentiation potential. MSCs can be obtained from the adult bone marrow (BMSCs), adipose tissue, umbilical cord, Wharton’s jelly, and the synovial membrane. BMSCs are currently the most studied and best characterized progenitor cells for cartilage engineering. Recently, a new, promising strategy for cartilage regeneration has been identified by isolating MSCs from the synovial fluid, with expansion over a short period of time, leading to the successful differentiation of the cells in chondrocytes (Matsukura et al. 2014). Notably, higher levels of synovial fluid-derived MSCs have been reported in the knee joint of patients with degenerated cartilage and OA. On the basis of their morphology and gene expression profiles, synovial fluid-derived MSCs are more similar to synovium-derived MSCs than to BMSCs (Sekiya et al. 2012).

**Figure 1:**
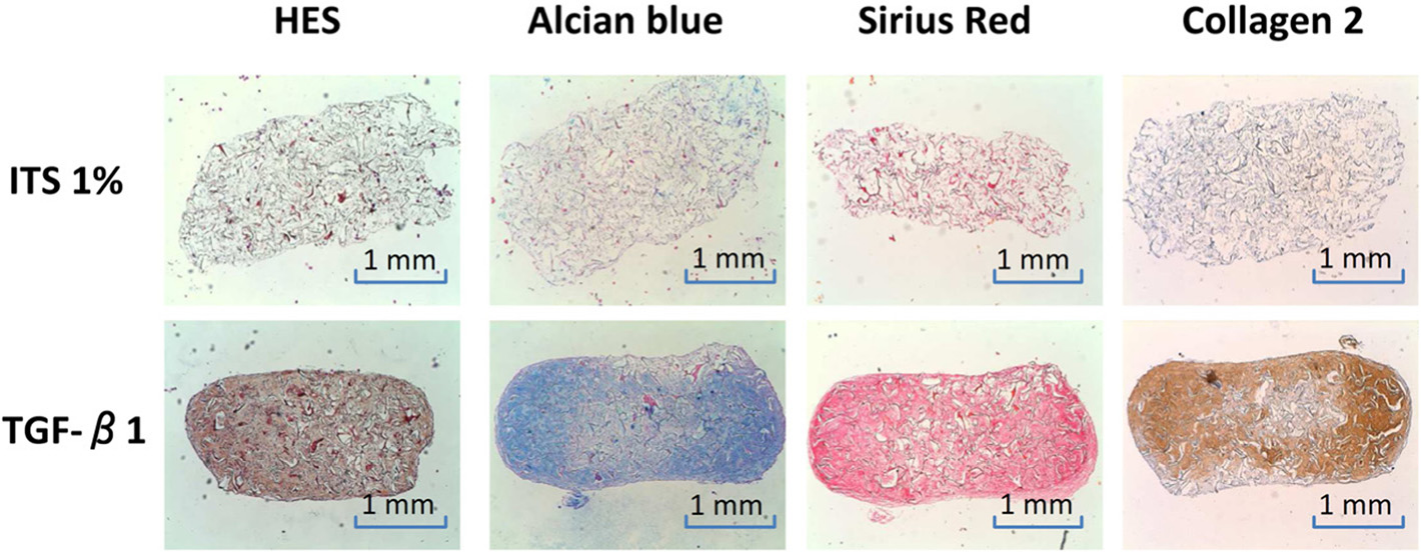
**Chondrogenic differentiation of human bone marrow-derived MSCs in collagen sponges after 28 days of culture in presence of TGF-β1** ***versus*** **defined chondrogenic ITS medium.** TGF-β1-induced matrix synthesis inside the sponge (HES: Hematoxylin-Eosine-Saffron) rich in proteoglycans (Alcian blue) and collagens (Sirius Red), particularly in type-II collagen.

Three important parameters apart from the origin of the transplantable cells may be used to maintain the chondrocyte phenotype and/or to promote chondrogenic differentiation: 1) the presence of growth or soluble factors, 2) the effects of mechanical loading, and 3) the stimulation by environmental factors such as hypoxia. The presence of growth or soluble factors (e.g. vitamins) in the culture medium is known to modulate the conditions of chondrocyte culture and chondrogenic differentiation. Members of the transforming growth factor beta (TGF-β) superfamily are good candidates to promote chondrogenesis (Figure 1) (Madry et al. 2014; Henrionnet et al. 2010). Addition of such factors, alone or in combination with others (bone morphogenetic proteins; fibroblast growth factors, i.e. FGFs) during the phase of chondrocyte expansion allows for a better maintenance of the chondrocyte phenotype and for an effective chondrogenic differentiation of MSCs (Perrier et al. 2011). Induction of stem cell differentiation by applying mechanical forces is another innovative concept in artificial tissue generation (Henrionnet et al. 2012). Hydrostatic pressure is a key component of the *in vivo* joint environment and has been shown to enhance the chondrogenesis of stem cells. It can act synergistically with growth factors to upregulate the expression of SOX9 (a key chondrogenic transcription factor) and the synthesis of cartilage-specific matrix molecules (proteoglycans, type-II collagen) while downregulating the expression of genes associated with terminal differentiation (type-X collagen) (Vinardell et al. 2012). *In vivo*, the cartilage and chondrocytes are exposed to low oxygen tension (2-7% saturation), contributing to the maintenance of the chondrocyte phenotype and to a tight control of the chondrogenic commitment and differentiation of various types of MSCs. MSC isolation and expansion under hypoxic conditions (3%) increases the ability of the cells to undergo a more robust chondrogenesis. Studies also suggest that hypoxia, like growth factors, may be a potential tool to control hypertrophic MSC differentiation (Studer et al. 2012). Of note, the majority of the studies available thus far are limited to combining one or two of these modulators while the concomitant effects of factors, loading, and hypoxia have not been documented yet although such a setup clearly represents the “real articular joint condition”.

### Non-invasive characterization of cartilaginous implants

In cartilage bioengineering, early detection according to the organization of the collagen network is a crucial step in the differentiation process of autologous cells seeded in a collagen biomaterial (matrix). To achieve a purpose-made biofunctionality, engineered cartilage needs to exhibit biochemical and physical properties similar to those of the native, hyaline cartilage. Additionally, early analysis of the amount of type-II collagen produced *versus* resident-matrix type-I collagen is a crucial step for non-destructive, preclinical implantation validation. The goal of our work was to develop a novel, non-invasive procedure that permits to characterize the collagen network in 3D constructs by Second Harmonic Generation (SHG) imaging (Stoltz et al. 2010).

To integrate the notion of multiscale imaging in real “clinical” live (non-sliced samples, sterile, non-invasive) analyses, a two-photon excitation laser was adapted to a macroscope optical way. To combine multidimensional fluorescence data (time and spatial), a new multimodality imaging (SHG-TCSPC) based on the SHG method was considered to detect especially collagen matrix and Time Correlated Single Photon Counting (TCSPC) method to contrast signal (auto-fluorescence background, SHG and immunolabelling staining) (McCourtie & Poxton 1990; Werkmeister et al. 2009). For both SHG-TCSPC optical systems (microscopy and macroscopy), an index F-SHG based on decay time response measured by TCSPC for respectively fluorescence (F) and SHG values was introduced. This index was previously defined in relation with the behavior of cells (F for proliferation; SHG for collagen synthesis) (Dumas et al. 2010).

The technique was validated using human BMSCs (hBMSCs) as a population of cells capable of chondrogenic differentiation when subjected to appropriate culture conditions (Kuroda & Dezawa 2014). hBMSCs were seeded in a scaffold (collagen sponge) that offers a high level of complexity, recapitulating the 3D organization of chondral tissue, and maintained in culture using the highly chondrogenic TGF-β1 growth factor. hBMSC-seeded 3D collagen sponges were processed to evaluate the effects of TGF-β1 upon type-II collagen expression in the extracellular matrix by macroscopic analysis of entire sponges (7-mm diameter) *versus* microscopic (histological) evaluation (5-μm thick sections). Similar increases in type-II collagen expression produced by the cells seeded in the constructs were noted after 28 days of culture with TGF-β1 using either microscopic or macroscopic tests. The levels achieved were always higher than in the absence of growth factor, regardless of the technique applied (Hematoxylin-Eosine-Saffron and Sirius red staining, immunolabelling, multiphoton imaging, TCSPC-SHG, F-SHG index).

**Figure 2:**
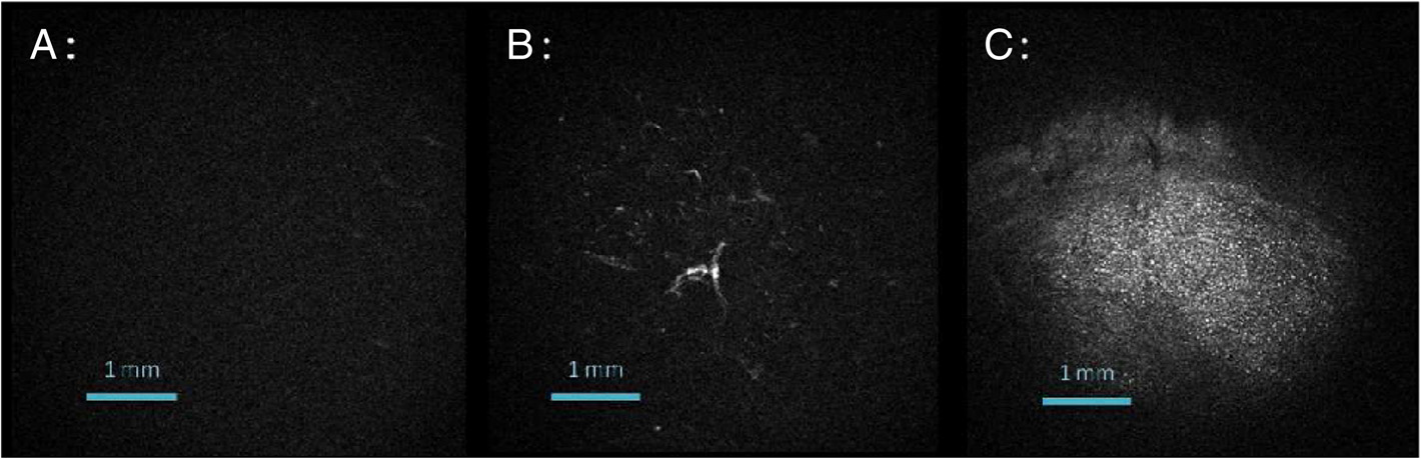
**Direct observation of three different scaffolds by TCSPC-SHG macroscopy for the detection of collagen as a harmonophore. (A)** Sponge without cells; **(B)** MSCs cultured for 28 days in sponges in the presence of ITS; **(C)** MSCs cultured for 28 days in sponges in the presence of TGF-β1.

Collagen network SHG imaging simplifies the interaction study between cells and collagen or molecules linked to collagen. Indeed, microscopy multiphoton allows to observe a first fluorescent marker via 2 photon-excitation-fluorescence and to visualize collagen without marking with SHG (Werkmeister et al. 2008). From a general point of view, such findings demonstrate the relevance of SHG microscopy to control the quality of a biomaterial in real time, without sacrifice, and especially to follow this individual evolution according to the patient’s cell capacity to differentiate into chondrocytes. Moreover, the possibility of detecting collagen as a harmonophore via TCSPC-SHG, without the need for an exogenous probe, may be advantageous to measure *ex vivo* cartilage degeneration (density and homogeneity of the collagen matrix). This technique may be used for the convenient and rapid screening of a large number of biomaterials simultaneously, noninvasively, and nondestructively, as a means to optimize the implant before grafting in a cartilage lesion (Figure 2). The next step will be to be able to discriminate between type-I and type-II collagens as previously described (Su et al. 2010).

Second Harmonic Generation is thus a sensitive technique for a “live” characterization of fibrillar collagens *versus* more conventional techniques. As it can be applied to many types of samples, SHG is robust, without additional manipulation among the studies using fluorescent markers. Despite its cost, it is so far the only procedure capable of defining the 3D organization of collagen in engineered tissues.

### Recombinant adeno-associated viral vectors as efficient tools for musculoskeletal gene therapy

Application of chondroreparative and chondroregenerative factors to sites of cartilage damage is an attractive approach to improve the quality of the healing response of cartilage to injury. Yet, a direct administration of recombinant molecules is impeded by their relatively short pharmacological half-lives (some minutes) due to the rapid clearance from the host. Approaches based on the transfer of genetic coding sequences have the advantage of allowing for the sustained production of a candidate agent in a desired location like in a cartilage lesion (Cucchiarini & Madry 2005).

**Figure 3:**
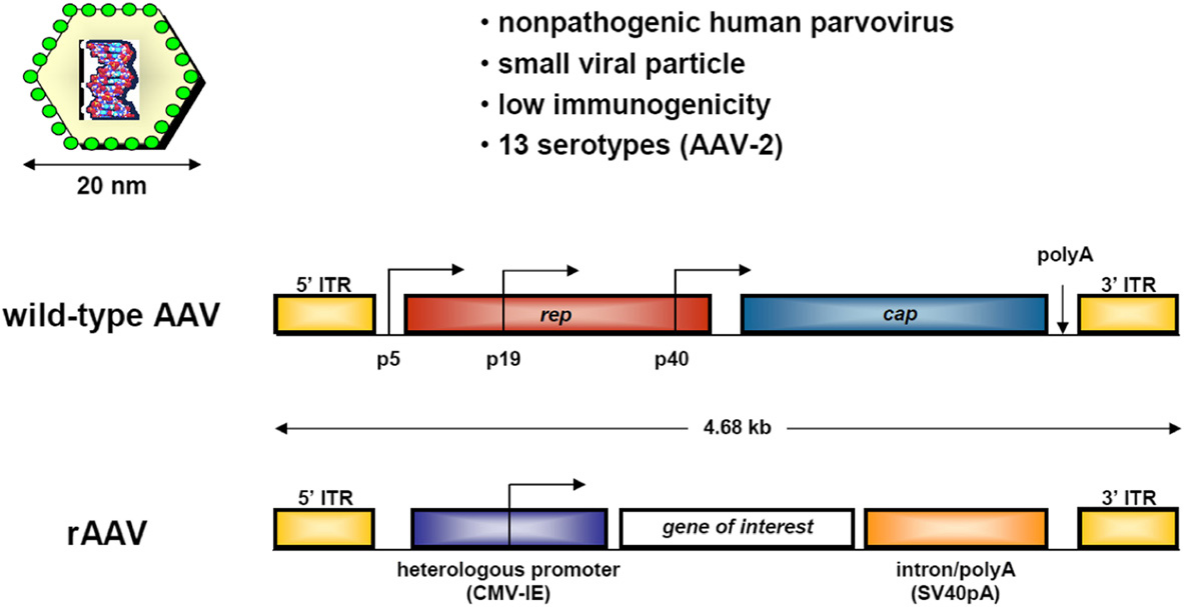
**Generation and characteristics of recombinant adeno-associated virus (rAAV) gene therapy vectors.**

Recombinant adeno-associated virus (rAAV) vectors are currently the best suited, clinically relevant gene delivery systems for human gene therapy and regenerative medicine as they are based on the non-pathogenic, replication defective adeno-associated virus (AAV) and have been reported for both their elevated gene transfer efficiencies and higher safety compared with other more classical classes of vectors (nonviral vectors, adenoviral, retro-/lenviral, or herpes simplex virus-derived vectors) (Frisch et al. 2014a; Cucchiarini et al. 2014; Madry & Cucchiarini 2013; Cucchiarini & Madry 2013; Madry et al. 2011; Cucchiarini & Madry 2005; Cucchiarini et al. 2003). This is mostly due to the fact that all AAV coding sequences can be removed from the recombinant vector genome and replaced by a transgene cassette, and that rAAV are mainly kept as highly stable episomal structures that can be expressed over extended periods of time without the need for integration in undesired (oncogenic) sites in the host genome (Figure 3).

Early work demonstrated the ability of rAAV vectors coding for reporter genes (*E. coli* beta-galactosidase gene; *Discosoma sp*. red fluorescent protein) to successfully modify normal and OA human adult articular chondrocytes in primary culture *in vitro*, in cartilage explant tissue *in situ*, and in sites of articular damage *in vivo* (Arai et al. 2000; Madry et al. 2003). In these systems, the transduction efficiencies exceeded 70%, resulting in reporter gene expression within cartilage explant tissue to a depth exceeding 450 μm that remained present for at least 150 days.

The benefits of applying various rAAV vectors to deliver growth (FGF-2; insulin-like growth factor I, i.e. IGF-I; TGF-β) and transcription (SOX9) factors in cartilage lesions in clinically relevant animal models of focal defects and of OA were next documented, allowing to improve the capacities of this tissue for stable repair for at least 4 months *in vivo* without development of a deleterious host immune response to the vectors or the transgene products (Cucchiarini & Madry 2014; Cucchiarini et al. 2013; Cucchiarini et al. 2005). Also of note, all these constructs were successful to transduce various human cells of the musculoskeletal system *in vitro* and *in situ*, including human normal and OA articular chondrocytes (Madry et al. 2003) and bone marrow-derived mesenchymal stem cells (hMSCs) (Cucchiarini et al. 2011; Stender et al. 2007). Such approaches led to the significant and durable remodelling of OA cartilage (at least 3 months) (Venkatesan et al. 2013; Weimer et al. 2012; Cucchiarini et al. 2009; Cucchiarini et al. 2007) and to enhance the chondrogenic differentiation of MSCs (Rey-Rico et al. 2014a; Frisch et al. 2014b; Frisch et al. 2014c; Venkatesan et al. 2012; Cucchiarini et al. 2011; Pagnotto et al. 2007) while delaying undesirable, premature chondrocyte hypertrophy and terminal differentiation (Cucchiarini et al. 2011; Venkatesan et al. 2012).

Altogether, rAAV vectors appear as particularly advantageous gene vehicles capable of promoting the effective, durable, and safe healing and remodelling of injured articular cartilage. The recent example of the market authorization of Glybera® (alipogene tiparvovec, an rAAV vector engineered to express the lipoprotein lipase in the muscles of patients with lipoprotein lipase deficiency) (Carpentier et al. 2012; Gaudet et al. 2012a; Gaudet et al. 2012b; Rip et al. 2005; Ross et al. 2004), the first commercially-approved gene therapy product in the West by the European Medicines Agency (EMA 2012) is thus a strong, motivating sign to develop clinically grade rAAV-based products for articular cartilage regenerative medicine.

### Evidence for an inflammatory and degradative role of the infrapatellar fat pad in OA

While early research in OA focused mostly on the articular cartilage, an innovating hypothesis has been proposed that may take into account 1) the well-established link between OA and obesity and 2) the changes in the whole OA joint, including synovium, bone, muscle, ligaments, joint capsule, and cartilage (Pottie et al. 2006). It is now well recognized that OA develops in the highly metabolic and inflammatory environment of adiposity. The sole role of biomechanical loading can not explain the increased risk for OA in non-weightbearing joints among overweight persons, and recent studies indicated that adiposity (rather than simply excess in body mass) is detrimental to the joint (Ding et al. 2008; Teichtahl et al. 2009; Toda et al. 1998; Wang et al. 2009). In obese subjects, the adipose tissue exhibits an aberrant secretion pattern. The enlarged adipose tissue is infiltrated with activated macrophages and several other types of inflammatory cells, leading to an increased production of proinflammatory adipokines (Lumeng et al. 2007).

As an articular adipose tissue, the infrapatellar fat pad (IFP) is an important candidate to become the focus of investigations that aim at further understanding the pathophysiology of OA (Clockaerts et al. 2010). Until recently, this extrasynovial but intraarticular tissue has been neglected even though it clearly releases growth factors, cytokines, and adipokines (Ushiyama et al. 2003). Work from our laboratory showed that in addition to the synovium, the IFP is an important source of adipokines in the joint, especially leptin and adiponectin (Presle et al. 2006). A crosstalk between adipocytes and other cells in the IFP may then regulate the cellular functions both in the cartilage and the synovium and promote articular changes associated with OA. Such work demonstrated a contribution of the IFP to local inflammation in knee OA and to cartilage degeneration through the release of cytokines and adipokines, in relation to the better characterized subcutaneous adipose tissue (scAT).

In these studies, specimens of cartilage, synovium, IFP, and scAT were obtained from OA patients undergoing total knee replacement surgery. Conditioned media were generated from cultured IFP and scAT explants, and cells (chondrocytes, synoviocytes) were isolated after sequential enzymatic digestion of the corresponding tissues. The data showed that the IFP released elevated amounts of leptin and adiponectin. Interestingly, the IFP from male OA patients exhibited similar secretory activity than the scAT, but the production of both adipokines differed between both adipose tissues for female OA patients. The IFP was the major source of adiponectin while the scAT released elevated levels of leptin. Besides, the conditioned media from IFP strongly induced the expression of microsomal prostaglandin E synthase-1 (mPGES-1) and cyclooxygenase-2 (COX-2) both in chondrocytes and synoviocytes. The mRNA level for inducible nitric oxide (NO) synthase (iNOS) was markedly increased in chondrocytes but not in synoviocytes. The expression of the genes encoding degradative enzymes (metalloproteinase 13, i.e. MMP13; ADAMS with thrombospondin motifs 4, i.e. ADAMTS-4) and to a lesser extent that of IGF-I was upregulated in the chondrocytes. In contrast, the conditioned media from the IFP did not stimulate the expression of TGF-β, aggrecan, or type-II collagen. Conditioned media from the IFP increased the production of PGE2 and NO and the activity of MMP13 in chondrocytes, stimulating also the release of PGE2 in culture supernatants from synoviocytes (Table 1). Similar effects were noted with the scAT but at different extents compared with the patient-matched IFP.

**Table 1:** **Effects of IFP-conditioned media on gene expression in patient-matched chondrocytes and synoviocytes**

**Genes**	**Chondrocytes**	**Synoviocytes**
iNOS	427.22 ± 123.76*	2.63 ± 0.45*
COX-2	57.39 ± 12.85*	362.75 ± 145*
mPGES-1	41.25 ± 7.51*	13.64 ± 1.82*
MMP13	18.29 ± 2.96*	ND
ADAMTS-4	86.64 ± 21.13*	ND
IGF-I	8.86 ± 2.04*	4.60 ± 2.64
TGF-β1	1.49 ± 0.28	1.09 ± 0.16
Type-II collagen	1.43 ± 0.19	ND
Aggrecan	1.27 ± 0.18	ND

The expression of inflammatory genes (iNOS, mPGES-1, COX-2) and of genes coding for matrix components (type-II collagen, aggrecan) or for factors involved in cartilage remodelling (MMP13, ADAMTS-4, IGF-I, TGF-β1) was analyzed in patient-matched cells (n = 20) by real-time quantitative PCR after 24 h of culture in paired conditioned medium. Data are expressed as means ± SEM of the mean over control values. *P ≤ 0.05 between IFP conditioned media-treated and control cells.

The data indicated that IFP is a particularly active organ with a major endocrine function, releasing notably leptin and adiponectin. The IFP and scAT exhibited different secretory patterns for adipokines in female OA patients. More importantly, the IFP may also be an important source of inflammation for chondrocytes and synoviocytes, thus contributing to articular changes associated with OA. Future studies on the identification of IFP-derived inflammatory mediators and on the characterization of infiltrating immune cells will be critical to understand the biology of this tissue.

## Discussion

Despite the availability of various options in the clinics, lesions to the articular cartilage remain a major unsolved problem due to the poor healing properties inherent to this avascular tissue. The development of new, effective therapeutic regimens is therefore critical to stimulate the intrinsic repair capacities of the cartilage. The use of basic science tools including cells, genes, and biomaterials that might be implanted in sites of cartilage lesions may open new avenues of clinically adapted research in orthopaedic surgery. Challenges that remain to advance the field of cartilage engineering include the choice of the best suited source of cells to undergo optimal chondrocyte differentiation without undesirable, premature chondrocyte hypertrophy and osseous metaplasia in dedicated scaffolds (Johnstone et al. 2013). Advances in high-resolution cartilage imaging greatly increased the ability to generate information on its 3D structure in a non-destructive fashion (Zehbe et al. 2010; Goebel et al. 2012; Goebel et al. 2014). Especially noteworthy is the recent availability of adapted experimental tools such as Second Harmonic Generation (SHG) imaging to perform a “live” structural characterization of cartilaginous constructs prior to implantation *in vivo*. In the field of gene therapy, the recent example of the market authorization of an rAAV vector-based gene medicine raises strong hopes for a clinical translation of this potent technology for musculoskeletal applications. Finally, the IFP has been identified as another particularly active organ involved in OA. It remains to be seen whether apart from its roles in OA, the IFP and in particular its associated mediators might be of value as targets for therapeutic interventions to enhance cartilage repair. Such advances in basic scientific and translational research in diverse fields of articular cartilage repair may have strong value to further develop improved clinical therapies (Madry 2014).

## Conclusion

In light of the new advancements in basic and applied experimental cartilage research, establishing a constant exchange of knowledge between scientists and clinicians appears to be the most adapted method to generate new, effective therapeutic options for an optimal treatment of articular cartilage lesions in patients.
